# Goal-Directed Fluid Therapy and major postoperative complications in elective craniotomy. A retrospective analysis of a before-after multicentric study

**DOI:** 10.1186/s12871-022-01962-5

**Published:** 2023-01-09

**Authors:** Morgan Le Guen, Amandine Le Gall-Salaun, Julien Josserand, Augustin Gaudin de Vilaine, Simon Viquesnel, Damien Muller, Bertrand Rozec, Kévin Buffenoir Billet, Raphaël Cinotti, the Société Française d’Anesthésie-Réanimation–SFAR Research Network, Amélie Yavchitz, Stéphanie Sigault, Aurélien Mazereaud, Lucilia Bezu, Maxime Léger, Jean-Noël Evain

**Affiliations:** 1 Department of Anesthesia, Hôpital Foch, 40 Rue Worth, 92150 Suresne, France; 2 Department of Anaesthesia and Critical Care, CHU Rennes, Hôpital Pontchaillou, 35000 Rennes, France; 3 Department of Anaesthesia, Hospital of Saint-Nazaire, 11 Boulevard George Charpak, 44600 Saint-Nazaire, France; 4 Department of Anaesthesia and Critical Care, CHU Nantes, Nantes Université, Hôpital Laennec, 44000 Nantes, France; 5 University of Nantes, CNRS INSERM, Institut du Thorax, 44000 Nantes, France; 6 Department of Neuro-Traumatology, CHU Nantes, Nantes Université, Hôtel Dieu, 44000 Nantes, France; 7 Department of Anaesthesia and Critical Care, CHU Nantes, Nantes Université, Hôtel Dieu, 44000 Nantes, France; 8 UMR 1246 SPHERE “MethodS in Patients-Centered Outcomes and HEalth Research”, University of Nantes, University of Tours, INSERM, IRS2 22 Boulevard Benoni Goulin, 44200 Nantes, France

**Keywords:** Craniotomy, Goal directed fluid therapy, Outcome, Postoperative complications

## Abstract

**Background:**

Goal-Directed Fluid Therapy (GDFT) is recommended to decrease major postoperative complications. However, data are lacking in intra-cranial neurosurgery.

**Methods:**

We evaluated the efficacy of a GDFT protocol in a before/after multi-centre study in patients undergoing elective intra-cranial surgery for brain tumour. Data were collected during 6 months in each period (before/after). GDFT was performed in high-risk patients: ASA score III/IV and/or preoperative Glasgow Coma Score (GCS) < 15 and/or history of brain tumour surgery and/or tumour greater size ≥ 35 mm and/or mid-line shift ≥ 3 mm and/or significant haemorrhagic risk. Major postoperative complication was a composite endpoint: re-intubation after surgery, a new onset of GCS < 15 after surgery, focal motor deficit, agitation, seizures, intra-cranial haemorrhage, stroke, intra-cranial hypertension, hospital-acquired related pneumonia, surgical site infection, cardiac arrythmia, invasive mechanical ventilation ≥ 48 h and in-hospital mortality.

**Results:**

From July 2018 to January 2021, 344 patients were included in 3 centers: 171 in the before and 173 in the after (GDFT) period. Thirty-six (21.1%) patients displayed a major postoperative complication in the Before period, and 50 (28.9%) in the After period (*p* = 0.1). In the propensity score analysis, we matched 48 patients in each period: 9 (18.8%) patients in the After period and 14 (29.2%) patients in the Before period displayed a major perioperative complication (*p* = 0.2). Sixty-two (35.8%) patients received GDFT in the After period, with great heterogeneity among centers (*p* < 0.05).

**Conclusions:**

In our before-after study, GDFT was not associated with a decrease in postoperative major complications in elective intra-cranial neurosurgery.

**Supplementary Information:**

The online version contains supplementary material available at 10.1186/s12871-022-01962-5.

## Background

Elective intra-cranial neurosurgery still bears a high rate of perioperative complications such as pneumonia, seizures, or intra-cranial bleeding and a high postoperative mortality rate [1]. The rate of major postoperative complications requiring management in a critical care unit also reaches up to 14.3% in recent cohorts [1]. There are little interventions that could reduce the rate of major postoperative complications in intra-cranial neurosurgery. Goal-Directed Fluid Therapy (GDFT) is currently recommended in major surgery to decrease the incidence of perioperative complications [2, 3]. This strategy has been well documented in abdominal and orthopaedic surgery in randomized-controlled studies [4]. GDFT decreases the incidence of thromboembolic events, postoperative wound infections, decreases hospital length of stay and costs [2]. Thus, the perioperative management of high-risk patients with a GDFT is recommended, with a high level of evidence [2, 3]. However, there is little evidence about the efficacy of GDFT in elective intra-cranial neurosurgery. We implemented a protocol of GDFT in patients undergoing elective craniotomy in 3 university hospitals in France. The goal of our study was to evaluate the efficacy of a GDFT on post-operative major complications, in a before-after multicentric study.

## Methods

This is a multicentre before-after study performed in 3 university hospital in France from July, 1^st^ 2018 to December 31^st^, 2021. The study was approved by an ethics committee (Groupe Nantais d’Éthique dans le Domaine de la Santé, N°8/01/2020). Patients received written information prior to participation. This was a retrospective analysis of prospective database available among centres. As the implementation of GDFT was part as a quality improvement in participating centres, IRB approval was not mandatory at the beginning of the process according to French regulations but was obtained for research purposes.

### Inclusion criteria

Patients above 18-years old were included in case of elective intra-cranial tumour surgery with craniotomy and in the presence of a risk factor of perioperative complication [1, 5]: ASA score III and IV, a preoperative Glasgow coma score (GCS) < 15, history of brain tumour intra-cranial surgery, tumour greater size ≥ 35 mm, mid-line shift ≥ 3 mm and a significant haemorrhagic risk (meningioma, tumour location).

### Exclusion criteria

Patients were excluded in case of the following criteria: < 18-years old, pregnant women, refusal to participation, urgent surgery and a GCS < 10 before surgery. Intra-cranial surgery for another indication than tumour: aneurysm clipping, abscess evacuation, hematoma surgery.

### Data collection

We collected age, gender, weight, height, ASA status, history of craniotomy, preoperative GCS, the use of antiepileptic drug and the use of corticosteroids (> 1 mg/kg) before surgery, the use of anti-hypertensive drugs. The following characteristics of brain tumour were collected: histology, greater size (mm), presence of a mid-line shift (≥ 3 mm), infra or supra-tentorial location, appreciation of the haemorrhagic risk. The following data during surgery were collected: position, type of hypnotic and morphine drugs, monitoring of sedation, invasive arterial pressure catheter, use of cardiac output monitoring, minimum and maximum of systolic and mean arterial pressure, volume of vascular expansion, use of norepinephrine, use of osmotherapy, intra-operative diuresis (mL), blood loss (mL) and transfusion, duration of surgery (mn). The following postoperative complications were recorded: need of re-intubation after surgery, a GCS < 15 after surgery, a focal motor deficit which did not exist before surgery, agitation requiring physical contention, seizures, intra-cranial haemorrhage, stroke, intra-cranial hypertension defined as intra-cranial pressure ≥ 25 mmHg and/or the need of barbiturates and/or the need of osmotherapy), health-care related pneumonia, surgical site infection or intra-cranial infection, cardiac arrythmia (fibrillation or flutter > 60 s), the need of a re-do neurosurgery because of complication, intensive care unit length of stay, in-hospital mortality.

### Implementation of the protocol

Patients were operated according to guidelines and local standards. Briefly, all patients underwent general anaesthesia for intra-cranial brain tumour surgery with either propofol or halogens. The level of sedation was not standardized and could be performed on-demand. Patients received either sufentanil or remifentanil according local standards. Peripheral vein access was established and an isotonic crystalloid solution 10 ml/kg before induction and 2–4 ml/kg/h intraoperatively was infused. Mean arterial pressure was set ≥ 60 mmHg. Central venous line could be added in case of a significant haemorrhagic risk, an infra-tentorial location or poor peripheral vein access. Mannitol was administered during surgery on demand of the neurosurgeon. Intraoperative normothermia was actively maintained with an air warming blanket. In all hospitals, patients were systematically discharged to the critical care unit.

Before the implementation of the protocol, the monitoring of cardiac output and GDFT were not standardized and were performed at the attending anaesthetist’s discretion. GDFT was performed in patients deemed at the highest risk of post-operative complications: ASA score III and IV and/or a preoperative Glasgow coma score (GCS) < 15 and/or history of brain tumour intra-cranial surgery and/or tumour greater size ≥ 35 mm and/or mid-line shift ≥ 3 mm and/or a significant haemorrhagic risk (meningioma, tumour location). Cardiac output monitoring could be performed either with an invasive or a non-invasive device. In the 3 centres, the devices available were the EV1000 Edwards® with either a Flotrac® or a Clearsight® and the MostCare Vygon®. In the GDFT protocol, once the device was set, we first administered 250 mL of fluid in order to define the stroke volume of reference (SV). If fluid responsiveness was not achieved, defined as a 10% SV increase [2], another 250 mL of fluid was administered to set the SV of reference. Vascular expansion (250 mL) was triggered in case of decreased Indexed Stroke Volume (≥ 10%) or mean arterial pressure < 65 mmHg. A Stroke Volume Variation increase ≥ 10% was used to assess the efficacy of vascular expansion. The use of static parameters and heart rate to perform vascular expansion was left at the attending physician’s discretion. EtCO2 was not considered in our centres to perform vascular expansion. When fluid responsiveness was not observed, continuous norepinephrine was administered to ensure a mean arterial pressure ≥ 65 mmHg.

A leader centre (N°2) elaborated the protocol and it was implemented in centre N°1 and N°3. In each centre the duration of the before and the after period was 6 months in each phase. The implementation of the protocol was performed in each centre during a period of 3 months, in order to train personal and master the protocol. Owing to the COVID-19 pandemic, some centres implemented the protocol after the 3 months of training. During this training period, no data were collected. Each centre decided of the timing of implementation of the protocol. For instance, in the leader centre N°2 the before period was performed between July and December 2018 and the after period was performed between April and October 2019; in centre N°1 and N°3, the before period was between June and November 2019, and between September 2019 and March 2020 respectively. In centre N°1 and N°3 the after period was between July and December 2020, and between August 2020 and January 2021.

### Primary outcome

The primary aim of the study was to evaluate the efficacy of a GDFT in patients undergoing elective intra-cranial surgery for a brain tumour on perioperative complications. The primary endpoint was a composite of major postoperative complications [1, 2]: need of re-intubation after surgery, the occurrence of a novel GCS < 15 after surgery which was not present before, a focal motor deficit which did not exist before surgery, agitation requiring physical contention, seizures, intra-cranial haemorrhage, stroke, intra-cranial hypertension defined as intra-cranial pressure ≥ 25 mmHg and/or the need of barbiturates and/or the need of osmotherapy), health-care related pneumonia, surgical site infection or intra-cranial infection, cardiac arrythmia (fibrillation or flutter > 60 s), the need of a re-do neurosurgery because of complication, invasive mechanical ventilation ≥ 48 h and in-hospital mortality. The presence of at least one complication was retained for the primary endpoint.

### Secondary outcomes

We also explored the differences regarding the management during surgery between patients from the Before and the After period. In the After period, we explored the rate of application of the protocol.

### Statistical analysis

Continuous data are expressed as median [interquartile] and nominal data as N(%). Univariate analysis was performed between patients “Before” and “After” the implementation of the protocol, according to the Student t-test and Chi2 test accordingly. Univariate analysis was performed in baseline demographic data, perioperative data, and post-operative data. Regarding the primary endpoint (occurrence of at least one major postoperative complications), the comparison between groups with a Chi2 test. In a recent multicentric cohort [1], 11% of patients were hospitalized in a critical care unit for more than 24 h, but 28% of patients displayed major postoperative complications as currently defined. Considering the OPTIMISE study and the effect of a GDFT in decreasing major postoperative complications in abdominal surgery [2], we expected to decrease the rate of postoperative complications in intracranial surgery from 28 to 23%, and 644 patients were needed in this study (322 patients per phase). Unfortunately, because of the COVID-19 pandemic, the implementation of the protocol was disrupted. Added to the difficulties regarding the availability of health-care professionals to apply the protocol in this period, the study was prematurely interrupted and was not re-started because of the ongoing pandemic. Moreover, 3 centers could not gather data during this period. Eventually the study was prematurely stopped after the inclusion of only 344 patients. We analyzed results between the “Before” and the “After” period. Since this study was observational, the allocation of GDFT was neither blinded nor randomly assigned and we used propensity-score matching to reduce the risk of bias, and assess the link of the intervention on the outcome [6]. Each patient treated in the intervention period was matched a control patient in the before period with a similar propensity score. Variables included in the propensity score model were selected from the baseline variables and the association between factors and cardiac output monitoring: age, ASA class, meningioma, hemorrhagic risk, the maximum size of brain tumor and the expected duration of surgery (> 2 h). Patients were matched according to the nearest neighbor approach within a caliper width of 0.1. To assess the balance of covariates between the two groups before and after propensity-score matching, mean standardized differences (MSD) were used. A mean standardized difference < 20% was considered to support the assumption of balance between groups [7]. In this matched sample, we compared the incidence of the composite endpoint. The level of significance was set at *p* < 0.05. Statistical analyses were performed with Rstudio® Version 1.0.153.

## Results

From July 2018 and January 2021, 344 patients were included. One hundred and seventy-one patients were included « Before» the implementation of GDFT and 173 were included « After» the implementation of the protocol. Patients were majorly women (200 (58.1%) patients), displayed an ASA class II (195 (56.7%) patients) and III (90 (26.2%) patients). One hundred and eight (31.4%) patients displayed chronic hypertension, and 156 (45.3%) had anti-epileptic drug before surgery. There were no significant differences in the baseline characteristics between the Before and After period (Table 1). Patients were majorly operated on for a meningioma (96 (28.8%) patients), a glioma/glioblastoma (84 (24.2%) patients) or a secondary brain tumor (102 (29.7%) patients). Again, there were no significant differences in the brain tumor’s characteristics between the 2 groups (Table 2).

**Table 1:** Baseline characteristics in the overall population

	Before *N* = 171	After *N* = 173	*P value*
Centre			< 0.05
1	46 (26.9%)	47 (27.2%)	
2	73 (42.7%)	112 (64.7%)	
3	52 (30.4%)	14 (8.1%)	
Gender Male/Female	73 (42.7%)/98 (57.3%)	71 (41%)/102 (59%)	0.8
Age	59 [48–69]	60 [49–69]	0.2
ASA			0.3
1	26 (15.2%)	29 (16.9%)	
2	97 (56.7%)	98 (57%)	
3	48 (28.1%)	42 (24.4%)	
4	0	3 (1.7%)	
GCS = 15	162 (94.7%)	164 (95.4%)	0.8
History of Craniotomy	27 (15.9%)	32 (18.7%)	0.5
Antiepileptic drug	83 (49.1%)	73 (42.2%)	0.2
Corticosteroids	68 (40.2%)	71 (42%)	0.7
Hypertension	50 (29.4%)	58 (33.5%)	0.4

*ASA* American Society of Anesthesiology, *GCS* Glasgow Coma Score

**Table 2:** Characteristics of brain tumour

	Before *N* = 171	After *N* = 173	*P value*
Histology			0.3
Meningioma	41 (24.1%)	58 (34.9%)	
Glioma/Glioblastoma	43 (25.3%)	41 (24.7%)	
Secondary tumour	58 (34.1%)	44 (26.5%)	
Tumour size ≥ 35 mm	92 (55.4%)	98 (60.5%)	0.3
Mid-line shift ≥ 3 mm	49 (29.5%)	60 (37.7)	0.1
Infra-tentorial location	42 (25%)	33 (19.2%)	0.2
Haemorrhagic risk	52 (54.7%)	81 (53.6%)	0.9

### Primary outcome

In the Before period, 36 (21.1%) patients displayed at least one major postoperative complication, whereas 50 (28.9%) patients displayed a major complication in the After period (p = 0.1). In the After period, patients displayed significantly more phlebitis or pulmonary embolism (5 (2.9%) vs 0, p = 0.03) or cardiac arrythmia (5 (2.9%) vs 0, p = 0.03). In the After period, more patients had a new onset of GCS < 15 (28 (16.2%) vs 11 (6.4%), p = 0.004). Table 3 displays the rates of postoperative complications in both groups. In the After period, only 62 (32.8%) patients received cardiac output monitoring. In the subset of patients receiving cardiac output monitoring and GDFT, only 13 (20.6%) patients displayed one complication (primary outcome).

**Table 3:** Data during surgery

	Before *N* = 171	After *N* = 173	*P value*
Supine position	134 (78.4%)	139 (80.4%)	0.6
Propofol	113 (66.1%)	156 (90.2%)	0.001
Sufentanil	81 (47.4%)	112 (64.7%)	0.001
Invasive arterial catheter	99 (57.9%)	148 (86.1%)	< 0.05
BIS monitoring	42 (24.6%)	46 (27.4%)	0.6
PPV monitoring	18 (10.8%)	129 (75.4%)	< 0.05
CO monitoring	1 (0.6%)	62 (35.8%)	< 0.05
SAP max (mmHg)	146 [126–166]	145 [132–164]	0.4
SAP min (mmHg)	80 [75–88]	85 [78–91]	0.1
MAP max (mmHg)	101 [87–113]	102 [91–116]	0.4
MAP min (mmHg)	60 [54–65]	59 [55–65]	0.9
Crystalloid (mL)	1500 [1250–2000]	1500 [1000–2000]	0.2
Norepinephrine	20 (11.7%)	67 (38.7%)	< 0.05
Ephedrine (mg)	9 [0–18]	9 [0–21]	0.03
Mannitol	29 (17.2%)	63 (37.5%)	< 0.05
Blood loss (mL)	250 [150–500]	200 [100–400]	0.007
Diuresis (mL)	200 [100–500]	325 [150–600]	0.3
Transfusion	7 (4.01%)	2 (1.2%)	0.1
Duration of surgery (mn)	137 [100–200]	158 [111–226]	0.5
Cranioscore (%)	10.1 [6.7–21.3]	11 [8.5–24.1]	0.1

*BIS* Bi-Spectral Index, *CO* Cardiac Output, *SAP* Systolic Arterial Pressure, *MAP* Mean Arterial Pressure, *PPV* Pulse Pressure Variation

In the propensity score analysis, we matched 48 patients who received GDFT in the After period with 48 patients from the Before period. In this analysis, 9 (18.8%) patients in the GDFT group displayed at least one post-operative complication and 14 (29.2%) patients in the group without GDFT displayed at least one complication (primary outcome) (*p* = 0.2).

### Secondary outcomes

During the After period and the implementation of GDFT, 62 (35.8%) patients received cardiac output monitoring. Patients in the After period received significantly more invasive arterial monitoring (148 (86.1%) vs 99 (57.9%), *p* < 0.05) and received more continuous norepinephrine infusion (67 (38.7%) vs 20 (11.7%), *p* < 0.05). Patients in the After period did not receive significantly different volume of crystalloids (1500 [1000–2000] vs 1500 [1250–2000], *p* = 0.2). Table 4 summarizes intra-operative data between the periods. There was a great heterogeneity between centers in the application of the protocol. In the first center, only 3 (6.4%) patients received GDFT, out of 47 who were deemed appropriate in this center, in the third center 22 (100%) out of 22 received GDFT and in the leader center (n°2) 46 (41%) out of 112 received GDFT.

**Table 4:** Post-operative complications

	Before *N* = 171	After *N* = 173	*P value*
Intubation/Reintubation	7 (4.1%)	8 (4.6%)	0.8
Health-related pneumonia	3 (1.8%)	3 (1.7%)	0.9
Variation of GCS	11 (6.4%)	28 (16.2%)	0.004
Agitation	2 (1.2%)	4 (2.3%)	0.4
Seizures	4 (2.3%)	6 (3.5%)	0.5
Intra-cranial haemorrhage	6 (3.5%)	13 (7.5%)	0.1
Stroke	3 (1.8%)	3 (1.8%)	0.9
Second neurosurgery	5 (2.9%)	7 (4.1%)	0.6
Intra-cranial hypertension	4 (2.3%)	8 (4.6%)	0.2
Phlebitis/Pulmonary embolism	0	5 (2.9%)	0.03
Atrial fibrillation	0	5 (2.9%)	0.03
SSI	2 (1.2%)	1 (0.6%)	0.5
ICU Readmission	7 (4.1%)	5 (2.9%)	0.5
Hospital LOS	7 [6-11]	7 [6-9]	0.1
In-hospital mortality	2 (1.2%)	3 (1.7%)	0.8
Primary outcome	36 (21.3%)	50 (28.9%)	0.1

The variation of GCS was defined as a GCS < 15 which was not present before surgery

*GCS* Glasgow Coma Score, *LOS* Length of Stay, *ICU* Intensive Care Unit, *SSI* Surgical Site Infection

## Discussion

In our multicenter before-after study in elective intra-cranial neurosurgery, the implementation of a GDFT protocol was not associated with a decrease in major postoperative complications. The COVID-19 pandemic has majorly impacted the implementation and application of our protocol.

GDFT has been documented for years in abdominal, vascular and orthopedics surgery [8–10]. Recently, the OPTIMISE study [2] was elaborated in 734 patients undergoing major abdominal surgery. The authors used a composite outcome which associated major postoperative complications. They failed to demonstrate an efficacy of GDFT in their original study, but the associated meta-analysis showed a significant decrease of major postoperative complications and hospital length of stay in patients receiving GDFT [2]. It is thus currently recommended to monitor cardiac output and perform GDFT in high-risk patients [3]. Intra-cranial neurosurgery remains a high-risk procedure, with a significant perioperative morbidity and mortality [1]. Patients undergoing intra-cranial neurosurgery usually have little co-morbidities which renders intra-cranial neurosurgery a high-risk procedure, compared to other surgery such as abdominal or cardiac surgery [11]. The impact of GDFT in this population has been little described. In a randomized-controlled trial performed in 145 patients, Luo et al. [12] found a significant decrease of postoperative complications as well as a significant decrease in the ICU length of stay. However, this was an open study and one cannot rule out a potential modification of practices in patients with GDFT. Moreover, the ICU length of stay in this study appears far from our current practices [1]. In another open randomized-controlled study in 80 patients evaluating two strategies of stroke volume in brain surgery, the authors found a decrease of ICU length of stay in the low stroke volume strategy group [13]. However, this study did not compare a GDFT versus a standard of care protocol. In our study, our GDFT strategy failed to demonstrate an efficacy on major perioperative complications.

Although protocols and standard of care are associated with improved outcomes in various settings, their implementation remains challenging in daily practice [14]. In multicenter before-after studies, the application of protocols can appear even more challenging, with only 15% of patients receiving the entire set of recommendations [15]. In our study, there was a wide variation of the implementation of the protocol among centers. Such phenomenon could significantly hamper the effect of a protocol. This raises the question of the relevance of before-after studies in a multicenter setting, where a leader center proposes to implement its protocol in other centers. Such strategy may not take into account other centers’ specificities, logistic issues, and could meet unexpected barriers and thus a low application.

Our study bears several limitations. First, it was an open observational study and there could have been a potential selection bias of patients with GDFT. The propensity score analysis could limit this bias. Second, there is a potential Hawthorne effect regarding the screening and declaration of postoperative complications in the After period. For instance, the number of patients with a new onset of GCS < 15 or phlebitis is significantly higher in the After period but there is currently little explanation for this finding. Third, we did not collect data about postoperative acute kidney injury which is commonly monitored in GDFT studies, as this outcome is poorly considered as a major outcome in patients after craniotomy. Fourth, some items such as the value of BIS or the dose of anesthetics which directly impact the hemodynamic response, were not collected. Finally, the COVID-19 pandemic has dramatically impacted this study: some centers which were willing to participate could not join, leading to less inclusions than expected. Even in unstressed periods, the implementation of a protocol is always challenging. The pandemic has totally disrupted its implementation, the availability of investigators, communication, leadership and recruitment of patients, resulting in an uneven application of the protocol. We decided to prematurely terminate the study, because of the remaining uncertainty of the evolution of the pandemic. Thus, the results should be cautiously interpreted especially the primary analysis.

## Conclusion

In our before-after multi-center study, GDFT was not associated with a decrease of major postoperative complications after elective intra-cranial neurosurgery. However, the COVID-19 pandemic has majorly disrupted the implementation process.

## Supplementary Information

**Additional file 1.**

## Data Availability

All data generated or analysed during this study are included in this published article as supplementary information files.

## Abbreviations

GDFT: Goal-Directed Fluid Therapy
ASA: American Society of Anesthesiology
GCS: Glasgow Coma Score
